# Influence of NR/MWCNT Blending on Rotor Metal Friction and Wear during Mixing Process

**DOI:** 10.3390/polym16162294

**Published:** 2024-08-14

**Authors:** Deshang Han, Quanzhong Zhang, Weifu Zhao, Changxia Liu, Lin Wang

**Affiliations:** 1 College of Transportation, Ludong University, Yantai 264025, China; handeshang@163.com (D.H.); jiaotongweifuzhao@163.com (W.Z.); liucxjiaotong@163.com (C.L.); ludongwanglin@163.com (L.W.); 2 Analysis and Testing Center, Ludong University, Yantai 264025, China

**Keywords:** MWCNTs, SiO_2_, rotor wear, mixing process, high-temperature steam

## Abstract

Mixing involves blending raw rubber or masticated rubber with additives using a rubber mixer, which is the most critical process in rubber production. The internal mixer, as the most important mixing equipment, experiences rotor wear during prolonged operation, affecting the gap between the mixer rotor and the chamber wall. This wear reduces mixing effectiveness, weakens filler dispersion, and ultimately impacts rubber performance. In recent years, as research on multi-walled carbon nanotubes (MWCNTs) and nanomaterials has deepened, their broad application prospects have become increasingly apparent. The objective of the present study is to understand and quantify rotor wear in rubber blends during the mixing process as influenced by multi-walled carbon nanotubes. This study found that with the increase in MWCNT content, the proportion of abrasive wear rises, while the proportion of corrosive wear decreases, leading to reduced overall wear. Compared to rubber without MWCNTs, the Payne effect decreased by 6.78%, 9.57%, 13.03%, 20.48%, and 26.06% with the addition of 1 phr, 3 phr, 5 phr, 7 phr, and 9 phr of MWCNTs, respectively. The friction coefficients between the rubber and metal increased by 6.31%, 8.57%, 25.43%, 39.31%, and 47.61%, while the metal wear rate decreased by 9.08%, 10.73%, 13.41%, 17.46%, and 25%. Conversely, the friction coefficients were reduced by 19.39%, 22.42%, 33.94%, 66.06%, and 76.36%.

## 1. Introduction

Research on the use of multi-walled carbon nanotubes (MWCNTs) as fillers in rubber materials has been a focal point [1,2]. Due to the manufacturing process and cost considerations, MWCNTs have more research value and application prospects in the rubber industry compared to graphene materials. Additionally, unlike traditional reinforcing agents such as carbon black, MWCNTs can retain more rubber molecular chains, forming denser network structures within the rubber matrix [3,4]. This impedes the deformation of rubber molecular chains, while frictional heat is quickly dissipated through MWCNTs, significantly contributing to the tear resistance, durability, and thermal conductivity of composite rubber materials [5,6].

Reports on using MWCNT fillers to enhance tire rubber performance are on the rise. From a material preparation perspective, the improvement in the tribological properties [7,8,9,10,11] of composite rubber by MWCNTs is mainly due to the entanglement of MWCNTs with rubber macromolecules, which increases tear strength and restricts the movement of rubber macromolecular chains. Gao et al. [12] suggested that wear resistance results from the competition between crosslink density and molecular chain flexibility, with imbalances leading to poor wear resistance. Molecular-scale studies have shown that the addition of MWCNTs reduces the bond and angle energies of rubber molecular chains, thereby lowering the friction coefficient. Variations in the amount and type of MWCNTs also affect their dispersion in composite rubber, influencing its tribological properties. Felhös et al. [13] found that the wear rate of hydrogenated nitrile rubber composites initially decreases and then increases with the content of MWCNTs. Kueseng et al. [14] studied the friction behavior of MWCNT-reinforced composites at different filler concentrations and found that at an MWCNT concentration of 0.2% (mass fraction), dispersion in the matrix is optimal, resulting in the lowest friction coefficient. However, as the concentration increases, the sliding contact area enlarges, causing a sharp rise in the friction coefficient to a peak value. Thus, increasing MWCNT content sometimes reduces tribological performance. Different friction pair forms also impact tribological properties. Foitzik et al. [15] observed that the friction coefficient gradually increases under sliding conditions, while it first decreases and then increases under rolling conditions.

As a typical one-dimensional material, the orientation of MWCNTs significantly affects the tensile and tear properties of carbon nanotube composite rubber, indirectly influencing its tribological performance. Gao et al. [16] filled natural rubber with aligned MWCNTs (A-MWCNTs) at different volume fractions and found that as the A-MWCNT content increased, the glass transition temperature of the natural rubber composites decreased, and thermal conductivity increased.

Sun et al. [17] developed a novel online imaging sensor utilizing magnetic deposition and fluid dispersion methods. This sensor can image both chain-like and isolated wear particles and holds promise for monitoring machine health and studying surface wear mechanisms, providing new insights into assessing rotor metal friction and wear during mixing processes. Wu et al. [18] proposed an online monitoring method for cutting tool conditions based on deep learning, which can accurately predict future flank wear based on collected multidimensional signals, avoiding the complexity of manual feature extraction. This method offers a basis for real-time wear measurement of mixer rotors in this study.

Research on carbon nanotube/rubber composites has been a hot topic in the rubber field in recent years. The production of these composites requires the use of internal mixers, during which the rotors inevitably experience wear. This paper investigates the effects of NR/MWCNT blending on rotor metal friction and wear during the mixing process.

## 2. Experiment

### 2.1. Formulation and Mixing Process

The formulation used in this experiment includes natural rubber (NR) 100 phr, silica (Silica 115MP) 30 phr, variable amounts (0 phr (C1), 1 phr (C2), 3 phr (C3), 5 phr (C4), 7 phr (C5), and 9 phr (C6)) of multi-walled carbon nanotubes (MWCNTs), ZnO 1.5 phr, stearic acid (SAD) 2 phr, silane coupling agent (TESPT) 5 phr, and diphenylguanidine (DPG) 1.5 phr. During vulcanization, accelerators sulfur (S) and N-cyclohexyl-2-benzothiazolesulfenamide (CZ) were added at 1.5 phr and 2 phr, respectively.

Natural rubber (NR) with a density of 0.92 g/cm^3^, a tensile strength of 20–30 MPa, and an elastic modulus of 0.05 GPa was obtained from Shanghai Cunsi Chemical Co., Ltd., (Shanghai, China). The anti-aging agent 4020, silica, zinc oxide (ZnO), stearic acid (SAD), accelerator CZ, and sulfur (S) were all industrial-grade products purchased from Tianjin Yiboruichem Co., Ltd., (Tianjin, China). Multi-walled carbon nanotubes (MWCNTs) with a diameter of 20–30 nm, a length of 10–30 μm, and a specific surface area of >110 m^2^/g were obtained from Sichuan Jia Materials Technology Co., Ltd., (Sichuan, China).

Set the cooling water temperature to 50 °C and start the internal mixer (SY-6212-A, Dongguan Shiyan Precision Instrument Co., Ltd., Dongguan, China). Add NR, MWCNTs, ZnO, SAD, TESPT, and DPG. After mixing for 60 s, add half of the Silica 115MP. Continue mixing for another 40 s before adding the remaining half of the Silica 115MP. Continue mixing for an additional 90 s. The total mixing time is set to 5 min. During the mixing process, monitor the temperature of the mixing chamber and open the upper piston for heat dissipation, maintaining the mixing temperature between 145 and 155 °C for 60 s. Then, extract the mixed compound and press it into smooth rubber samples using a two-roll mill (SHK-C202, Suzhou Jianzhuo Instrument Technology Co., Ltd., Suzhou, China). The rubber samples were cut into test specimens using a friction and wear testing machine (Swiss CSM- Friction and Wear Tester, Tribometer Co., Peseux, Switzerland).

### 2.2. Testing Methods

**Figure 1:**
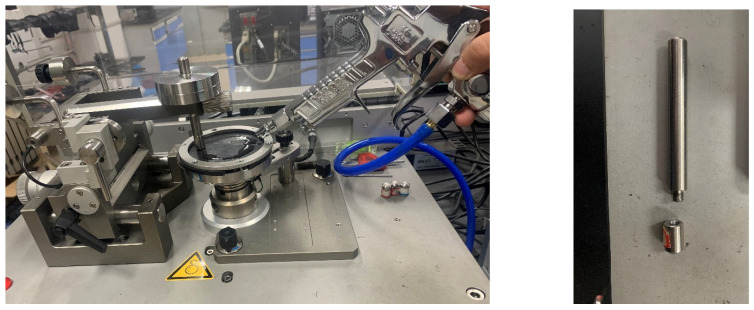
Photograph of CSM wear experiment equipment.

**Figure 2:**
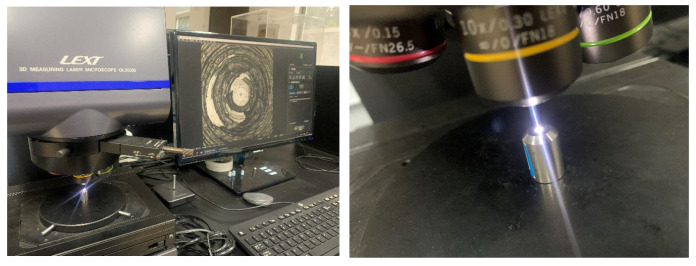
Olympus three-dimensional scanner.

(1) Payne Effect: Strain sweeps were conducted on six different mixed compounds using the rubber processing performance analyzer (MDR 3000, MONTECH, Buchen, Germany). The testing conditions included a scanning frequency of 1 Hz, a scanning range from 0.28% to 40%, and a temperature of 60 °C [19,20]. Curves depicting the dynamic modulus (G′) versus strain were generated. The Payne effect, characterized by a sharp decline in the dynamic modulus (G′) of filled rubber as strain increases, indicates the quality of filler dispersion; poorer dispersion leads to a more pronounced Payne effect [21,22].

(2) Silanization Reaction Index: The silanization reaction index was measured with the MDR 3000 rubber processing performance analyzer [23,24]. This index serves as a crucial indicator of the extent of silane modification in silica, with higher values signifying a greater degree of silanization and enhanced overall performance of the rubber compound [25].

(3) Friction and Wear Test: Friction and wear tests were executed using the CSM friction and wear tester. After calibration, the test conditions were established with an applied pressure of 5 N, a rotational speed of 70 r/min, and a test duration of 120 min. To examine the wear on the internal mixer’s rotor, the metal wear head was made of the same material as the rotor. Prior research has shown that rotor wear is most severe in the final stage of the experiment; hence, the CSM temperature was set to 150 °C. The uniformly surfaced blended rubber obtained from the open mixer was cut into cylindrical rubber specimens with a diameter of 100 mm and a thickness of 8 mm. The CSM is shown in Figure 1.

(4) Three-Dimensional Morphology Observation: A 3D laser measuring microscope (LEXT OLS5000, Olympus Corporation, Tokyo, Japan) was utilized to observe the metal surface morphology. The wear volume was determined by measuring the reduction in the volume of the metal wear head. OLS5000 is shown in Figure 2.

(5) Dispersion Testing: Dispersion was assessed using a dispersion analyzer (DisperGRADER, Alpha Technologies, Akron, OH, USA), with dispersion values obtained in accordance with ASTM D7723 standards [26].

## 3. Mechanism of Silanization Reaction

**Figure 3:**
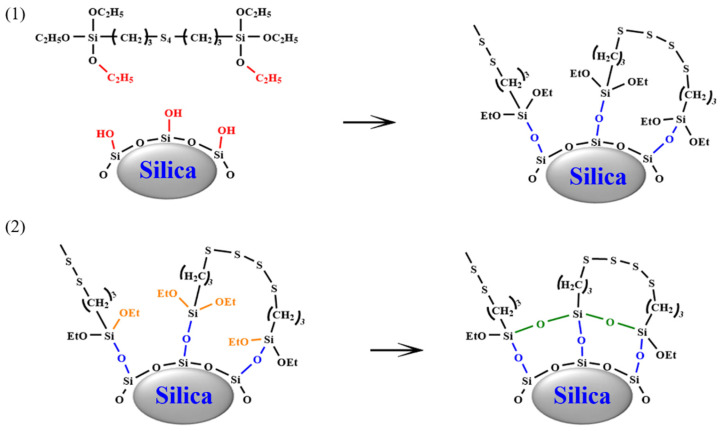
The silanization reaction: (1) initial reaction; and (2) subsequent reaction.

The process of silanization starts when TESPT (triethoxysilylpropyl tetrasulfide) attaches itself to the silica’s surface. During this phase, the silane’s alkoxy groups interact with the hydroxyl groups on the silica, resulting in silanization [27,28]. This procedure comprises two distinct stages: an initial reaction and a subsequent reaction. In the first stage, two key reactions take place: the direct alcohol condensation between TESPT’s alkoxy groups and silica’s silanol groups, and the dehydration condensation involving the hydrolyzed alkoxy groups of TESPT and the silanol groups [29,30]. The second stage features the condensation of neighboring TESPT molecules that have chemically bonded to the silica surface [31]. Figure 3 depicts the silanization process.

Studies show that wear intensifies significantly during the final mixing phase, where elevated temperatures prevail and the internal mixer remains sealed. In such a high-temperature environment, steam cannot vent out, making it essential to account for corrosion wear from high-temperature steam when examining rotor end-face friction and wear. Nevertheless, taking apart the internal mixer’s rotor end face proves to be exceedingly challenging, and it is unfeasible to gauge the steam mass produced. In the friction tests carried out using the CSM friction and wear testing apparatus, high-temperature steam was proportionally applied to the rubber–metal surface mix to replicate the internal mixer conditions based on the extent of the silanization reaction. During these experiments, a steam generator (ZT-2588S, Zhiteng Company, Taiwan, China) was employed to dispense the high-temperature steam.

## 4. Experimental Results

### 4.1. Filler Dispersion Analysis

#### 4.1.1. Payne Effect

**Figure 4:**
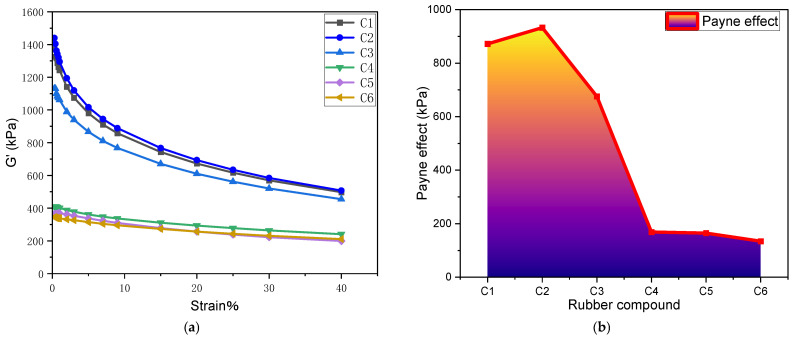
(**a**) Stress-strain curve; (**b**) Payne effect.

Figure 4 and Table 1 display the Payne effect for compounds with varying MWCNT contents, depicting dynamic modulus (G′) versus strain. As the amount of MWCNTs increases, the initial decline in the Payne effect is evident. Compared to rubber without MWCNTs, the compound containing 1 phr of MWCNTs exhibited a 6.92% increase in the Payne effect. In contrast, the Payne effect was reduced by 22.52%, 80.64%, 81.11%, and 84.59% in the compounds with 3 phr, 5 phr, 7 phr, and 9 phr of MWCNTs, respectively. Specifically, the inclusion of 1 phr and 3 phr of MWCNTs shows a slight decrease in the Payne effect compared to the MWCNT-free compound. However, a notable increase in the Payne effect is observed upon adding 5 phr of MWCNTs, whereas increasing the MWCNT content to 7 phr results in only a marginal change.

This highlights that a small quantity of MWCNTs minimally affects filler dispersion, but the addition of 5 phr of MWCNTs significantly impedes filler dispersion, leading to a marked rise in the Payne effect. Incorporating MWCNTs diminishes filler dispersion, impacting silica dispersion negatively, weakening the silanization reaction, and promoting silica aggregation.

#### 4.1.2. Dispersion Comparison

**Figure 5:**
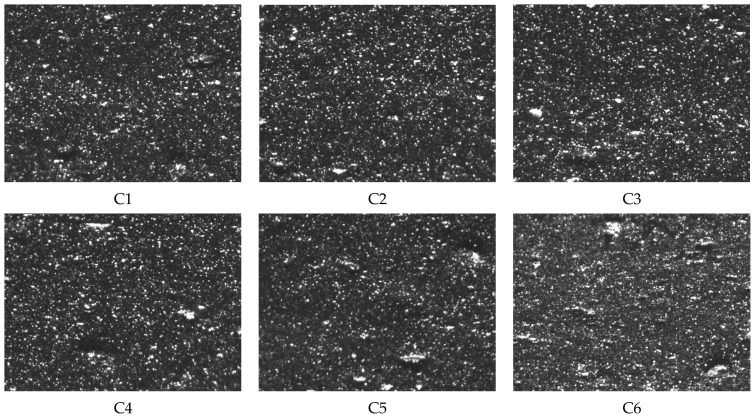
Dispersion images of mixed compounds with different CNT contents (C1, C2, C3, C4, C5, and C6).

Figure 5 and Table 2 demonstrate the silica dispersion in mixed compounds featuring different MWCNT contents. Compared to rubber without MWCNTs, the Payne effect was reduced by 6.78%, 9.57%, 13.03%, 20.48%, and 26.06% with the incorporation of 1 phr, 3 phr, 5 phr, 7 phr, and 9 phr of MWCNTs, respectively. With increasing MWCNT content, the dispersion of silica gradually worsens. In compound C1, which lacks MWCNTs, silica aggregates are fewer and smaller. Conversely, the addition of MWCNTs amplifies both the size and quantity of silica aggregates. Compound C6, containing 9 phr of MWCNTs, exhibits significantly larger and more abundant silica aggregates compared to C1.

### 4.2. Wear Analysis

#### 4.2.1. Silanization Reaction Index

**Figure 6:**
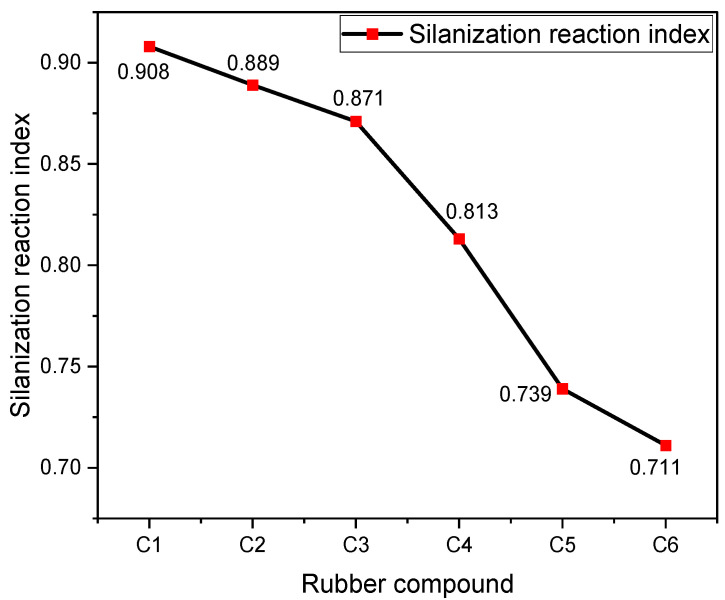
Silanization reaction index.

In silica-filled rubber compounds, the silanization reaction index serves as a measure of the degree of silanization, indicating the extent of bonding between silica and rubber molecules. A higher index suggests a more thorough silanization reaction and enhanced silica dispersion within the formulation. Moreover, this index can indicate the proportion of water produced as a byproduct during the silanization process. Figure 6 illustrates the silanization reaction index measured for six distinct mixed compounds using a rubber processing analyzer.

Figure 6 illustrates a progressive decrease in the silanization reaction index with increasing MWCNT content, consistent with the trends observed in the Payne effect and dispersion outcomes. The generation of high-temperature steam, a byproduct of the silanization reaction, presents a corrosive risk to metals, which must be accounted for in friction and wear assessments. These tests involved spraying high-temperature steam at 150 °C in ratios corresponding to the silanization reaction index values of 1:0.98:0.96:0.89:0.81:0.78.

#### 4.2.2. Coefficient of Friction

**Figure 7:**
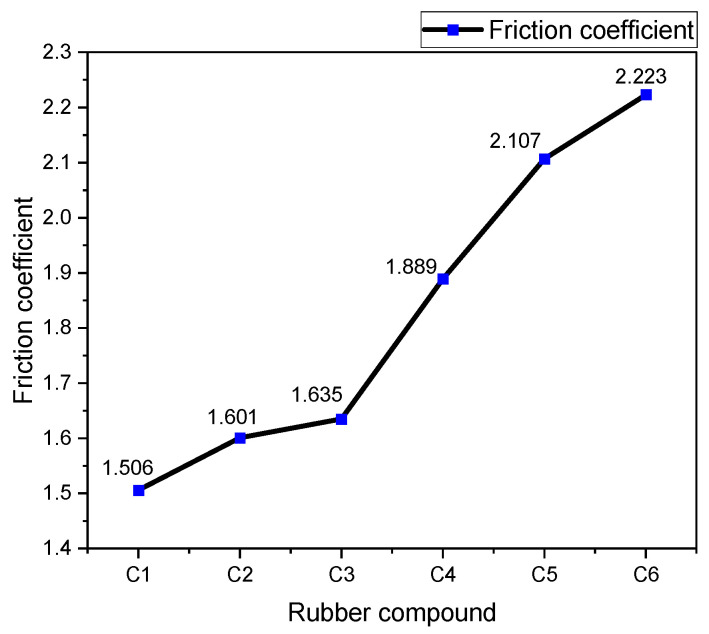
Average friction coefficient.

Based on the experimental findings depicted in Figure 7, variations in the coefficient of friction between rubber blends containing different MWCNT contents and metals were observed. It can be observed from Figure 7 that the friction coefficients between the rubber and metal increased by 6.31%, 8.57%, 25.43%, 39.31%, and 47.61% with the incorporation of 1 phr, 3 phr, 5 phr, 7 phr, and 9 phr of MWCNTs, respectively, compared to the rubber without MWCNTs.

The rubber blend exhibited the lowest coefficient of friction against metals when devoid of MWCNTs. However, as MWCNT content increased, the coefficient of friction gradually escalated. This trend is attributed to the deterioration of silica particle dispersion caused by the addition of MWCNTs, resulting in increased silica aggregation within the rubber blend and consequently higher friction coefficients.

Due to their distinct structural and physical characteristics, MWCNTs not only impede the interaction between silica molecules and silane coupling agents but also significantly enhance resistance to metal movement, thereby further amplifying the coefficient of friction between the rubber blend and metals. In conclusion, augmenting MWCNT content noticeably enhances the coefficient of friction between the rubber blend and metals.

#### 4.2.3. Morphology of Metal Surface before and after Friction

**Figure 8:**
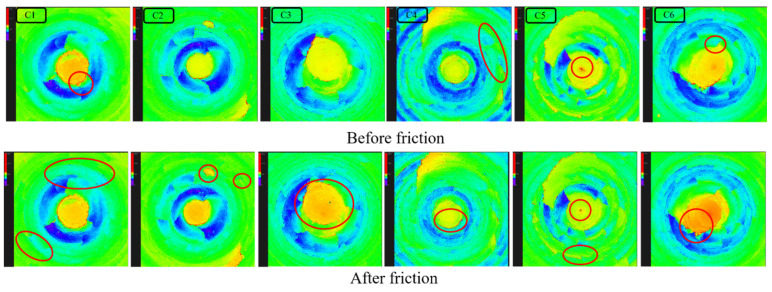
Metal surface morphology before and after friction between rubber compounds with different MWCNT contents and metal.

Comparing the images in Figure 8 (C1), significant darkened areas appeared on the metal surface post-friction, indicating an expansion trend in surface pits, suggesting severe wear. During the blending process without the addition of MWCNTs, a hindrance to SiO_2_ molecules by MWCNTs was absent, allowing for ample silanization reactions, and fewer aggregates of silica remained. Without MWCNT encapsulation, silica aggregates could directly contact the metal. Due to their high hardness, silica aggregates primarily caused abrasive wear on the metal surface, resulting in considerable wear when no MWCNTs were added to the rubber blend.

Contrasting with Figure 8 (C2), minimal contour changes were observed on the metal surface pre- and post-friction, with few darkened areas. MWCNTs exhibited some hindrance to SiO_2_ molecules, reducing the extent of silanization reactions. While MWCNTs hindered the movement of SiO_2_ molecules, their spatial structure also partially encapsulated some silica aggregates, preventing direct contact with the metal. Thus, adding one part of MWCNTs to the rubber blend reduced wear on the metal.

In Figure 8 (C3), the metal surface showed numerous fine scratches post-friction, with fewer darkened areas and minimal changes in pit morphology. The addition of three parts of MWCNTs during blending hindered their interaction with SiO_2_ molecules, further reducing the extent of silanization reactions. Despite SiO_2_ molecules tending to aggregate, the spatial structure of MWCNTs obstructed their movement, resulting in more silica aggregates that did not directly contact the metal. As a result, the rubber blend containing three parts of MWCNTs exhibited a continued reduction in metal wear.

Figure 8 (C4–C6) displayed relatively fewer scratches on the metal surface after friction, accompanied by minimal changes in surface contours and flattened pits. With increasing amounts of MWCNTs, their hindrance to SiO_2_ molecules intensified, further diminishing silanization reactions. Due to their hexagonal planar cylindrical structure and high toughness, MWCNTs acted as a medium for certain silica aggregates to reach the metal surface. Their unique spatial arrangement effectively encapsulated these silica aggregates, thereby reducing abrasive wear on the metal.

#### 4.2.4. Metal Wear Volume

**Figure 9:**
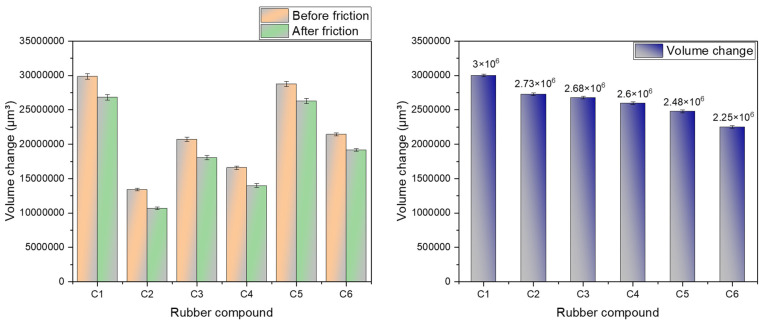
Metal wear volume.

Figure 9 and Table 3 depict the volume of metal wear observed from friction and wear experiments performed using a metal grinder head. Measurements were taken multiple times using a three-dimensional profilometer and averaged. The wear process of the silica-filled rubber blend on metal surfaces encompasses both abrasive and corrosive mechanisms. The progression of silanization reactions results in water generation, and the presence of high-temperature steam accelerates metal corrosion during wear. The data collected in this experiment represent the volume of metal wear under conditions involving high-temperature steam spraying, covering both abrasive and corrosive wear volumes.

Compared to rubber without MWCNTs, the addition of 1 phr, 3 phr, 5 phr, 7 phr, and 9 phr of MWCNTs reduced the wear rate of the metal by 9.08%, 10.73%, 13.41%, 17.46%, and 25%, respectively. During the blending process without MWCNTs, there is no interference from MWCNTs with SiO_2_ molecules, allowing for silanization reactions to proceed unhindered, resulting in fewer residual silica aggregates. Without MWCNT encapsulation, silica aggregates can directly contact the metal, leading to abrasive wear primarily due to their high hardness. Consequently, the rubber blend without MWCNTs exhibits the highest wear volume on metal.

Incorporating MWCNTs partially impedes SiO_2_ molecules in the rubber blend, thereby reducing silanization reactions. MWCNTs restrict the movement of SiO_2_ molecules and partially encase silica aggregates due to their unique spatial structure, preventing direct metal contact. Consequently, the rubber blend with one part of MWCNTs shows reduced metal wear.

Increasing MWCNT content intensifies their hindrance to SiO_2_ molecules, further reducing silanization reactions. With their hexagonal planar cylindrical structure and exceptional toughness, MWCNTs effectively encapsulate some silica aggregates, facilitating their interaction with the metal. While silica aggregates typically cause abrasive wear on metal surfaces due to their hardness, the wear volume decreases significantly when MWCNTs mediate contact between silica aggregates and metal. Therefore, increasing MWCNT content continues to decrease the wear volume of the rubber blend on metal.

#### 4.2.5. Proportion of Corrosive Wear and Abrasive Wear

**Figure 10:**
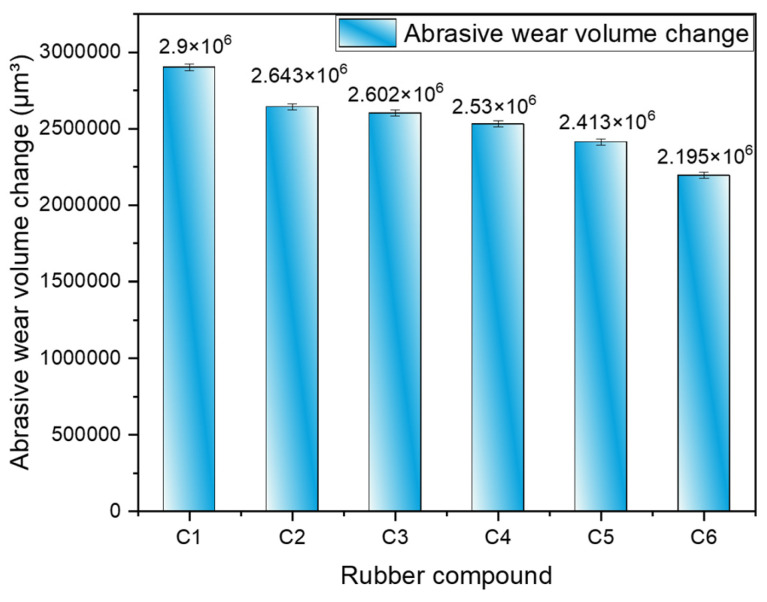
Volume of metal wear without high-temperature steam spraying.

To establish a baseline, friction and wear experiments were conducted using a silica-filled rubber blend without spraying high-temperature steam, serving as a control, and subjected to CSM friction and wear tests. Figure 10 and Table 4 illustrate the volume loss of the metal specimens after these experiments. Compared to rubber without MWCNTs, the addition of 1 phr, 3 phr, 5 phr, 7 phr, and 9 phr of MWCNTs reduced the abrasive wear of the metal by 8.9%, 10.29%, 12.79%, 16.8%, and 24.34%, respectively. In this scenario, high-temperature steam was not applied, thereby eliminating corrosive wear. Hence, the observed wear was purely abrasive. Introducing high-temperature steam results in both abrasive and corrosive wear occurring concurrently. By comparing the wear under conditions with and without high-temperature steam spraying across six repeated experiments and averaging the results, the proportion of abrasive wear to corrosive wear can be determined.

**Figure 11:**
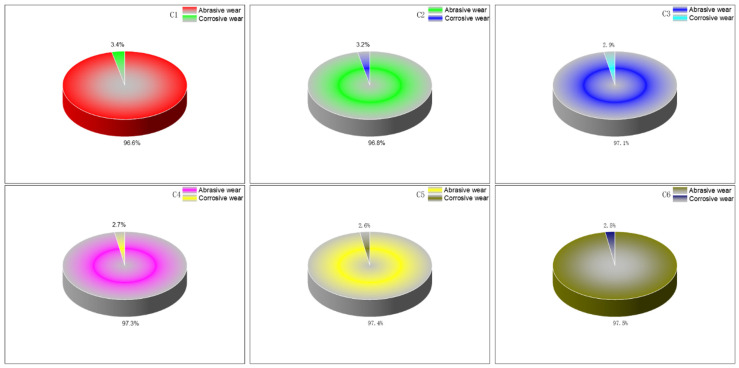
Wear ratio.

From Figure 11, it is evident that as the number of MWCNTs increases, the proportion of corrosive wear gradually decreases. When there are no MWCNTs, corrosive wear occupies the highest proportion. As the amount of MWCNTs increases, their hindrance to SiO_2_ molecules intensifies, further reducing the extent of silanization reactions. Silanization reactions generate high-temperature steam, and the decreased extent of these reactions during blending leads to reduced production of high-temperature steam, which contributes to corrosive wear. Therefore, increasing MWCNT content correlates with reduced corrosive wear.

MWCNTs possess a hexagonal planar cylindrical structure with exceptionally high toughness and flexibility. This unique spatial arrangement enables MWCNTs to effectively encapsulate some silica aggregates, facilitating their interaction with the metal. Due to their toughness and flexibility, MWCNTs cause minimal wear on the metal surface. As MWCNTs act as a medium for silica aggregates to contact the metal, the wear of the rubber blend on the metal decreases progressively with increasing MWCNT content.

#### 4.2.6. Roughness Changes before and after Friction

**Figure 12:**
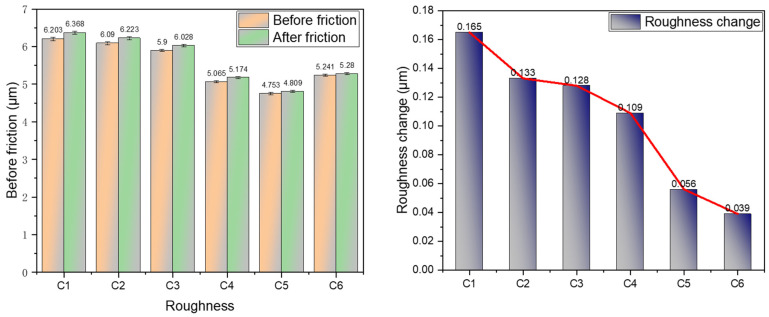
Metal surface roughness.

Figure 12 illustrates the variation in surface roughness of the metal grinder head before and after friction. Compared to rubber without MWCNTs, the friction coefficients between the rubber and the metal were reduced by 19.39%, 22.42%, 33.94%, 66.06%, and 76.36% with the incorporation of 1 phr, 3 phr, 5 phr, 7 phr, and 9 phr of MWCNTs, respectively. This study reveals that as the number of MWCNTs increases, the magnitude of surface roughness variation decreases. This reduction is attributed to the unique spatial structure of MWCNTs, which effectively encapsulate some silica aggregates, facilitating their interaction with the metal. With their high toughness and flexibility, MWCNTs contribute minimally to wear on the metal surface. As MWCNTs serve as a medium for silica aggregates to interact with the metal, the variation in metal surface roughness decreases with increasing MWCNT content.

## 5. Conclusions

Studies indicate that increasing the content of multi-walled carbon nanotubes (MWCNTs) during blending reduces the silanization reaction index, worsening silica dispersibility and promoting aggregate formation. However, this surprisingly leads to a decrease in metal wear. This study also found that with increasing MWCNT content, abrasive wear proportionally increases while corrosive wear decreases, resulting in reduced overall wear. Compared to rubber without MWCNTs, the Payne effect decreased by 6.78%, 9.57%, 13.03%, 20.48%, and 26.06% with the addition of 1 phr, 3 phr, 5 phr, 7 phr, and 9 phr of MWCNTs, respectively. The friction coefficients between rubber and metal increased by 6.31%, 8.57%, 25.43%, 39.31%, and 47.61%, while the metal wear rate decreased by 9.08%, 10.73%, 13.41%, 17.46%, and 25%. Conversely, the friction coefficients decreased by 19.39%, 22.42%, 33.94%, 66.06%, and 76.36%. Overall, this study has significant implications for reducing internal mixer wear, ensuring production efficiency, extending the lifespan of internal mixers, and maintaining the quality of rubber products.

## Figures and Tables

**Table 1:** Payne effect.

Rubber	C1	C2	C3	C4	C5	C6
Payne Effect (kPa)	872.17	932.55	675.74	168.89	164.73	134.41

**Table 2:** Dispersion values of mixed compounds with different CNT contents.

Rubber Compound	C1	C2	C3	C4	C5	C6
Dispersion	7.52	7.01	6.8	6.54	5.98	5.56

**Table 3:** Metal wear volume.

Rubber Compound	Before Friction	After Friction	Volume Change
C1	29,840,232.73	26,837,912.37	3,002,320.358
C2	13,426,579.44	10,696,922.25	2,729,657.189
C3	20,724,015.27	18,043,812.31	2,680,202.955
C4	16,598,935.15	13,999,310.09	2,599,625.051
C5	28,768,615.15	26,290,460.59	2,478,154.554
C6	21,440,262.44	19,188,570.74	2,251,691.703

**Table 4:** Volume of metal wear without high-temperature steam spraying.

Rubber Compound	Abrasive Wear Volume Change
C1	2,900,841.93
C2	2,642,581.125
C3	2,602,477.069
C4	2,529,955.1
C5	2,413,474.72
C6	2,194,723.903

## Data Availability

The original contributions presented in the study are included in the article, further inquiries can be directed to the corresponding author.
